# Clinical Signs at Diagnosis and Comorbidities in a Large Cohort of Patients with Lipedema in Spain

**DOI:** 10.3390/biomedicines13123049

**Published:** 2025-12-11

**Authors:** Jose Luis Simarro, Sandro Michelini, Miguel Andrés-Gasco, Alberto Lebrero García, Desirée Ortega Abad, José Margalejo Lombardo, Julian Buj Vargas, Jesús Tomás Sanchéz-Costa, María Auxiliadora Martín Martínez

**Affiliations:** 1 Instituto del Lipedema y de la Mujer, Calle de Modesto Lafuente, 45, 28003 Madrid, Spain; miguel.andresgasco@gmail.com (M.A.-G.); albertolebrerogarcia@gmail.com (A.L.G.); desire.ortega@institutolipedema.com (D.O.A.); pepemargalejo@gmail.com (J.M.L.); julian.buj@institutolipedema.com (J.B.V.); 2 Institute san Giovanni Battista Hospital, Via Luigi Ercole Morselli, 13, 00148 Rome, Italy; sandromichelini2018@gmail.com; 3 Investigación en Salud y Calidad Asistencial (INSyCA), 28860 Madrid, Spain; jesustsanchez@insyca.es (J.T.S.-C.); mariaamartin@insyca.es (M.A.M.M.)

**Keywords:** lipedema, diagnosis, signs and symptoms, morbidity

## Abstract

**Background/Objectives**: Lipedema is a chronic disorder that affects almost exclusively women and is characterized by bilateral, symmetrical accumulation of subcutaneous fat, typically in the buttocks, hips, and lower limbs, and in some cases the arms. The primary objective of this study was to describe the clinical and anatomical manifestations of lipedema, together with the associated physical and psychological comorbidities, in a large Spanish cohort. **Methods**: Descriptive study of 1803 patients aged ≥ 17 years who attended two clinics in Spain between January 2022 and November 2024. **Results**: The mean age was 42.9 years (SD: 11.3), and 60.6% of patients were diagnosed during their reproductive years. The mean body mass index was 28.6 (SD: 6.2), and 87.6% presented a gynoid fat distribution. A total of 46.6% were classified as Schingale stage IV or V. The most frequent comorbidities were chronic low-grade inflammatory alterations and connective tissue damage. Particularly suspected high intestinal permeability (99%), bilateral trochanteric pain region (97.4%), iliotibial band involvement, and ligamentous hyperlaxity (95.8%). Thyroid disorders, inflammatory ovarian dysfunction, and psychological impairment were also common. **Conclusions**: Lipedema is a complex condition that extends beyond lower-limb adipose tissue and is associated with multiple comorbidities. This study also presents a novel approach to clinical assessment that may help physicians gain a deeper understanding of this pathology and formulate etiological hypotheses that will need to be tested.

## 1. Introduction

Lipedema is a chronic disorder that predominantly affects women and is characterized by a bilateral and symmetrical increase in the volume of appendicular subcutaneous adipose tissue, with a nodular and fibrotic appearance, most frequently in the buttocks, hips and lower limbs [1]. In approximately 30% of cases, the upper limbs may also be affected [2,3,4]. Although lipedema was first described by Allen and Hines in 1940 [5], Lipedema was proposed as a disease by the World Health Organization (WHO) [6] in May 2018 and was officially accepted as such on 1 January 2022, as an independent clinical entity in the International Classification of Diseases (EF02.2; ICD-11).

This condition remains poorly understood and is often confused with other clinical entities such as lymphedema, phlebedema, obesity, lipodystrophy, obesity-induced lymphedema (OIL), and Madelung’s disease [7,8,9]. A recent review conducted in 2025 [4] highlights the persistent lack of knowledge regarding the management of this disorder and underscores the urgent need for high-quality clinical trials to improve understanding and to explore optimal medical and surgical therapeutic options for affected patients. This knowledge gap contributes to significant delays in diagnosis, high rates of unnecessary consultations with other specialists, deterioration in quality of life, and a substantial psychological and emotional impact on patients [10].

Data on the prevalence of this disorder are very limited and underestimated. In Spain, such information is unavailable, whereas in the United States, prevalence data in children indicate rates of up to 6.5% [3]. In Germany, the frequency of this condition has also been investigated, with reported prevalence ranging from 7% to 9.7% [2].

A limitation in diagnostics and patient care is the absence of validated diagnostic criteria or a reliable and readily accessible biomarker for lipedema, which renders the diagnosis fundamentally clinical and dependent on the clinician’s experience.

Coinciding with the WHO’s designation of lipedema as a disease in 2022 ICD 11, a literature review was published outlining the clinical features, diagnosis, and management of this condition [9]. The review emphasizes the importance of a thorough anamnesis to establish disproportionate fat distribution, the limited impact of weight loss on fat distribution, pain, tenderness to touch, and the lack of pain relief with limb elevation. Following the anamnesis, a comprehensive physical examination should be performed to determine the affected sites (thigh, leg, arm, and/or forearm) and to assess additional criteria such as tenderness on palpation and the presence of distal fat over the knee tendons. Since then, no large-scale studies have been published to confirm the presence of these criteria or to evaluate the distribution of their frequencies.

In addition to diagnostic criteria, a crucial aspect of this disorder is the associated comorbidities and impact on quality of life and mental health. A recent study by Luta X et al. demonstrated that pain affected nearly 90% (87.9%) of patients in their cohort, and poor physical and mental well-being was reported in 71.5% and 67.4% of patients, respectively [11]. Women with lipedema face not only physical symptoms but also high levels of social stigmatization and emotional challenges due to a lack of understanding by healthcare professionals [12]. Many patients report feelings of frustration, low self-esteem, and anxiety resulting from the absence of effective treatment options and the misperception of their condition as obesity [13,14]. These challenges are amplified in patients with more advanced stages of the disease, who often perceive that they are not taken seriously and are only offered advice on diet and lifestyle due to misdiagnosis as obesity or failure to consider a lipedema diagnosis [15].

Lipedema shows strong familial aggregation and likely autosomal-dominant inheritance with sex limitation. Early pedigree studies supported a genetic basis, later strengthened by candidate-gene evidence—most notably a missense variant in *AKR1C1* impacting progesterone metabolism in a multigenerational family—and by genome-wide signals implicating loci related to adipose/vascular biology (*VEGFA*, *GRB14*–*COBLL1*). Together, these data indicate a heritable, polygenic/heterogeneous architecture consistent with clinical clustering in women [16,17,18,19].

In 2016, the first clinical guideline was published in the Netherlands, establishing a series of clinical criteria for the diagnosis of lipedema, as well as an attempt to define a therapeutic algorithm [20]. In addition to diagnostic criteria, the guideline also defined a “basic set” of disease-specific criteria and clinimetric measures to consistently identify disease-related disability and impairments in daily functioning. To further advance understanding of the clinical signs and symptoms that characterize lipedema and distribution in affected patients. We set out to conduct a study whose main objective is to describe the signs, symptoms, and comorbidities in one of the largest lipedema cohorts in the world.

## 2. Methods

**Figure 1:**
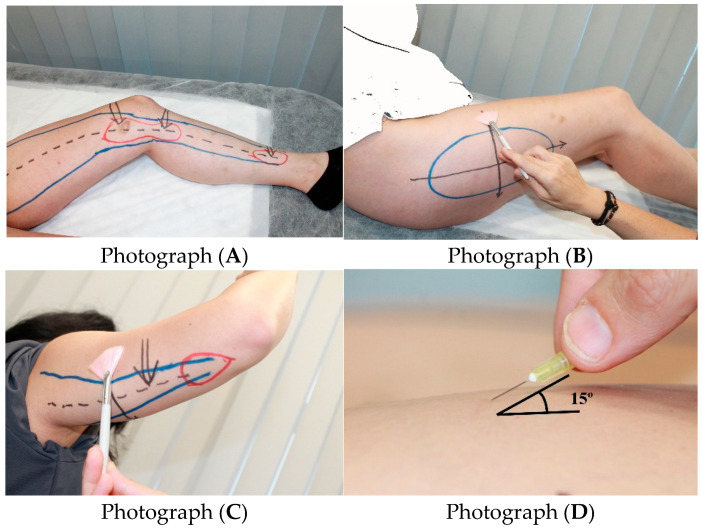
(Photographs (**A**–**D**)). Physical examination of lipedema: Simarro’s tender points, painful areas, the “false prick” sign, and loss of fine sensation.

A retrospective, observational, descriptive study based on the review of medical records of patients over 17 years of age with a clinical diagnosis of lipedema who attended the Instituto del Lipedema y de la Mujer clinics in Madrid and Barcelona between January 2022 and November 2024. As part of routine clinical practice during the diagnostic visit, patients underwent physical examinations and assessments, including multifrequency segmental bioelectrical impedance analysis (TANITA 480 MA) to evaluate fat mass, muscle mass, and body water both overall and individually for each of the five body segments (limbs and trunk/abdomen) [21], as well as bicipital skinfold caliper measurements. The following were evaluated: tender points, painful areas, pseudo-pinch sign, partial fine sensory loss at the pertrochanteric region, and neuropathic pain. Figure 1. The clinical judgment was based on the clinical experience of the professional with more than 17 years of care for patients with lipedema and on the diagnostic criteria collected in the first Dutch guide [20] and the literature review by Redondo Galán et al. [9].

**Photograph A**: The discontinuous black lines show the pathway of the saphenous nerve. The space enclosed within the blue line is the great saphenous vein compartment (GSphC). The areas marked in red are the areas positive for the “false prick” sign, and the black arrows indicate the Simarro points that are painful upon deep pinching.

**Photograph B**: The pertrochanteric area shows a loss of fine sensitivity. The pathway is marked by black lines using a fine brush. The patient notices a loss of fine sensitivity when entering the indicated area and recovers it upon exiting.

**Photograph C**: The discontinuous black lines refer to the pathway of the Nervus cutaneus brachii posterior, and the space within the blue line corresponds to the posterior brachial compartment (PBC). The space within the red line refers to the area positive for the “false prick” sign, and the black arrows correspond to the area painful upon superficial pinching. The loss of fine sensitivity is explored by passing a fine brush along the black line, and the patient notes a loss of fine sensitivity when entering the indicated area and recovery upon exiting.

**Photograph D**: Angle at which the sign of the “false prick” sign should be used within the areas marked with a red line. A cutaneous pin stimulus test was performed by pressing a sterile needle at a ~15° angle on the skin without piercing it. In areas of localized hyperesthesia, patients typically perceived this as a painful pinprick sensation—termed a ‘false positive prick sign’—reflecting nociceptive hypersensitivity in saphenous territories, a characteristic finding in lipedema and unrelated to allergy testing.

The following comorbidities or clinical phenomena were recorded: suspected high intestinal permeability assessed with an ad hoc questionnaire (Table S1) [22,23,24,25,26,27,28,29,30,31,32,33] followed a robust and rigorous methodology through a review of the scientific literature and a formal expert consensus (from 17 years of clinical practice and with >4000 women diagnosed with lipedema in our center); ligamentous hyperlaxity based on the Beighton test (Table S2) [34]; inflammatory ovarian dysfunction (Table S3) [35,36,37]; thyroid disease as determined by ultrasound; allergies, particularly to nickel; and eating disorders assessed using the validated Spanish versions of the Eating Attitudes Test-40 (EAT-40) (Table S4) [38] and the SCOFF questionnaire (Table S5) [39]. The inflammatory ovarian dysfunction was assessment with the Inflammatory Ovarian Dysfunction Index (IODI), an ad hoc questionnaire developed after many years of clinical observation in more than 4000 women with lipedema.

### 2.1. Variables and Operational Definitions

Sociodemographic variables were collected, including sex and age, as well as date of lipedema diagnosis. Anthropometric variables included weight (kg), height (cm), body mass index (BMI; weight (kg)/height (cm)^2^) and its categorization (normal weight [18.5–24.9], overweight I [25–27.9], overweight II [28–29.9], obesity type I [30–34.9], type II [35–39.9], type III [40–49.9], type IV [≥50], and underweight [<18.5]). Body composition was assessed by segmental multifrequency bioimpedance analysis (BIA), which directly quantifies total and regional fat mass. This method provides a more accurate distinction between fat and lean tissue than indirect indices such as BMI or WHtR. For descriptive purposes, the waist-to-hip ratio (WHR) was calculated following WHO criteria (<0.85 for gynoid, ≥0.85 for android pattern). The waist-to-hip ratio (WHR) was also recorded as an indicator of body fat distribution, classified as android pattern (predominant fat accumulation in the abdominal region, thorax, and upper body) or gynoid pattern (fat accumulation in the hips, thighs, and buttocks). During the physical examination, deep pressure pain was assessed in both the proximal and distal portions of the Great Saphenous Compartment (GSphC), in addition to deep pressure pain in the Small Saphenous Compartment (SSphC), and superficial pressure pain in the Posterior Brachial Compartment (PBC) (Figure 1). Specific tender points were assessed along the GSphC: (1) deep medial supracondylar, (2) deep medial infracondylar, (3) deep medial supramalleolar, and in the superficial supra-olecranon area of the distal PBC (yes/no) (Figure 1). The false prick sign was explored at two points along the GSphC and at one point in the PBC, as well as fine sensory loss at the pertrochanteric region, the Kaposi-Stemmer sign [40], the presence of supramalleolar edema with and without pitting, and distal pretibial edema with and without pitting (Figure 1).

Among comorbidities, the suspicion of high intestinal permeability was assessed using a 26-item ad hoc questionnaire (Table S1) [22,23,24,25,26,27,28,29,30,31,32,33]. The development of this questionnaire followed a robust and rigorous methodology through a review of the scientific literature and a formal expert consensus (from 17 years of clinical practice and with more 4000 women diagnosed with lipedema in our center). The cut-off score for identifying patients with suspected high intestinal permeability was 16 (see Supplementary Materials). Ligamentous hyperlaxity was determined using the Beighton score (0–9) [34] (see Supplementary Materials). The presence of bilateral trochanteric pain region, iliotibial band involvement (tensor fasciae latae), recurrent episodes of ankle twisting without sprain, associated with mechanical instability, and other comorbidities were also evaluated. Iliotibial band (ITB) involvement was evaluated with the patient in lateral decubitus and both legs extended, applying continuous longitudinal pressure from the greater trochanter to the lateral femoral condyle. A positive finding—bilateral pain along the ITB—was interpreted as a functional manifestation of ligamentous hyperlaxity, reflecting fascial distension and altered postural load rather than a localized mechanical lesion. These included clinically confirmed nickel skin allergy, thyroid disease as assessed by ultrasound (classified as echogenicity alterations, multinodular goiter, multinodular thyroid, or thyroid nodules), and inflammatory ovarian dysfunction, assessed using a four-item ad hoc questionnaire [35,36,37] (Table S3), in which the condition was diagnosed with three positive responses. Pain in paratibial and/or posterior tibial perforator veins was also recorded (yes/no) tested by deep, selective palpation at the site where the perforator vein traverses the muscular fascia. Eating disorders were assessed with the SCOFF questionnaire (0–5) [39] (Table S5) and the Eating Attitude Test-40 (EAT-40) (0–120) [38] (see Supplementary Materials). Fine sensory loss or tactile hypoesthesia at the pertrochanteric region was evaluated using a pine brush (Figure 1).

Segmental multifrequency bioelectrical impedance analyses were performed, from which the following parameters were obtained: total body fat mass (BFM) (kg), percentage of body fat to total weight (%), fat mass of the right lower limb (kg), percentage of fat mass relative to total mass of the right lower limb (%), fat mass of the left lower limb (kg), and percentage of fat mass relative to total mass of the left lower limb (%).

To classify patients with lipedema according to the involvement of body segments, the Schingale classification was applied [40]: type I, type II, type III, type IV, and type V (see Supplementary Materials). To classify patients based on adipose tissue involvement and skin texture, the Schmeller classification was used [2]: stage/grade I, stage/grade II, and stage/grade III (see Supplementary Materials). In addition, affected areas of lipedema were assessed clinically, by ultrasound, and by elastographic evaluation of the GSphC, divided into proximal and distal segments. Strain elastography was used for this purpose (Table 1). As an ultrasound marker of lymphatic involvement, the presence of pretibial (stone-paved sign) [41] was evaluated in the distal tibia region (see Supplementary Materials).

All patients underwent Doppler ultrasound with particular attention to the great saphenous vein (GSV) and its perforator veins. The presence of saphenous insufficiency was assessed. Even in patients without insufficiency or signs of perforator damage, such as a diameter greater than 3.5 mm or bidirectional flow, selective digital pressure was performed at the site where the perforator vein crossed the deep (muscular) fascia, and the presence or absence of pain during this maneuver was recorded.

The sample size of this study was based on convenience, including all patients who met the inclusion criteria and attended the Instituto del Lipedema y de la Mujer clinics during the study period.

All patients included in the study provided written informed consent for the use of their data for research and scientific dissemination during their diagnostic visit. The study was approved by the Regional Research Ethics Committee for Medicinal Products of the Community of Madrid in April 2025 (Code: LIPE-2024-01).

### 2.2. Statistical Analysis

A descriptive analysis was performed for sociodemographic and clinical variables. Normally distributed numerical variables were expressed as means and standard deviations (SD), while non-normally distributed variables were reported as medians and interquartile ranges (IQR, 25th–75th percentile). Associations between saphenous compartment involvement, stone-paved sign, age, weight, obesity type, and fat distribution were analyzed using bivariate Pearson correlations. Results are presented as Pearson correlation coefficients (r) with the corresponding level of statistical significance (*p*-value). Correlations were considered moderate for r values between 0.3 and 0.5 and strong for r values between 0.5 and 0.9.

All statistical analyses were performed using SPSS version 26.

## 3. Results

A total of 1846 patients were initially included in the study, of whom 43 were excluded from the analysis (2 men, 12 patients with incorrect age records, and 29 without a clinical diagnosis of lipedema), leaving a total of 1803 patients.

### 3.1. General Descriptive Data and Clinical Signs of Lipedema on Physical Examination

The mean age of patients was 42.9 years (SD: 11.3), ranging from 18 to 94 years. Of the cohort, 60.6% (1093) were of reproductive age (18–45 years), and 25.9% (467) were in the perimenopausal or menopausal age range (45–55 years). Regarding anthropometric parameters, the mean weight was 75 kg (SD: 16.4), mean height was 162 cm (SD: 6.3), and mean BMI was 28.6 (SD: 6.2). Overall, 31.9% (576) of patients were of normal weight, while 5.4% (98) had morbid or extreme obesity. In terms of body fat distribution, 87.8% (1583) exhibited a gynoid pattern.

Total BFM was measured via bioelectrical impedance in 1775 patients, with a mean of 27.7% (SD: 11.8) and a mean fat percentage relative to total body weight of 34.4% (SD: 9.8). Analysis of fat percentage by anatomical segments showed no significant differences between the left and right sides, but significant differences were observed between upper and lower body segments (Table 2).

On physical examination, pressure-induced pain was more prevalent in the lower limbs than in the upper limbs. Deep pressure pain in the proximal and distal thirds of the GSphC was present in 98.7% (1780) and 98.4% (1775) of patients, respectively. Similarly, deep pressure pain in the SSphC was observed in 96.4% (1738) of patients, while superficial pressure pain in the PBC was confirmed in 80% (1443) of patients. Regarding specific tender points (medial supracondylar and infracondylar, medial supramalleolar, and supra-olecranon), they were positive in nearly all patients. The pseudo-pinch sign at the medial supracondylar, medial supramalleolar, and supra-olecranon sites was present in 98.8% (1781), 97.1% (1750), and 81.8% (1474) of patients, respectively.

Fine sensory loss at the pertrochanteric region was only assessed in 195 patients and was positive in 90.3% (176) of them. The Kaposi-Stemmer sign was positive in 7.7% (138) of patients and indeterminate in 3.5% (63).

The presence of edema was also evaluated. Medial supramalleolar edema with pitting was very rare. In contrast, pretibial distal edema was present in 88.4% (259) of cases, and with pitting, generally grade 1, observed in a much lower percentage (57.8%). These signs could only be assessed in 293 and 270 patients, respectively. Medial distal supramalleolar edema was, with pitting edema generally grade 1, evaluated in a smaller subset of patients (n = 256) and was positive in 89.5% of them (Table 2).

Associations between saphenous compartment involvement, the pseudo-pinch sign, and fine sensory loss at the pertrochanteric region with age, weight, obesity type, and fat distribution pattern were weak (r < 0.3). In contrast, strong correlations (r ≥ 0.5) were observed between deep pressure pain in the saphenous compartment, tender points, and pseudo-pinch signs, as shown in Table 3.

### 3.2. Lipedema Classification and Diagnosis by Anatomical Region Based on Clinical, Ultrasound, and Elastography Findings

Based on anatomical location (Schingale classification), which could be determined in 1796 patients, nearly half exhibited lipedema affecting both arms and legs: 42.8% (768) were type IV and 3.8% (68) were type V. When analyzed according to adipose tissue structure and skin texture, 82.2% (1473) were classified as stage/grade I or II. Regarding the diagnosis of lipedema in the hips and abdomen, it was made almost entirely on clinical grounds (99.2%; 1787). Elastographic involvement of the GSphC was nearly 100% in the proximal half. When this proximal half was subdivided, involvement was 99.3% in the upper region and 97.7% in the lower region, whereas elastographic involvement in the distal half of the GSphC was markedly lower. Pretibial paved-stone sing tissue on ultrasound was observed in 47.2% (851) of patients with lower calf involvement, whereas it was infrequently detected in other anatomical regions (Table 4).

Pretibial stone-paved sign tissue below the ankle showed a moderate correlation with pretibial stone-paved sign tissue in the upper calf (r = 0.41; *p* < 0.001). Pretibial stone-paved sign tissue on ultrasound in the lower calf was moderately correlated with more extensive lipedema according to the Schingale classification (r = 0.376; *p* < 0.001). Furthermore, patients with higher body weight tended to exhibit more extensive lipedema (r = 0.487; *p* < 0.001) and higher stages according to adipose tissue involvement and skin texture (r = 0.541; *p* < 0.001) (Table 5).

### 3.3. Comorbidities and Lipedema

The most frequent comorbidities in patients with lipedema (Table 6) were suspected high intestinal permeability (99%; 1785) and symptoms related to ligamentous hyperlaxity (95.8%; 1726), including bilateral trochanteric pain region (97.4%; 1753) and iliotibial band involvement (96.6%; 1737). The mean Beighton score (0–9) was 7.5 (SD: 1.6). Endocrine disorders, notably inflammatory ovarian dysfunction and thyroid disease, were present in 76% (1368) and 59.5% (1073) of patients, respectively. Among the 533 patients who underwent thyroid ultrasound to classify the type of involvement, 473 (88.7%) were found to have thyroid nodules. Nickel skin allergy was assessed in 130 patients, of whom 88 (67.7%) were positive. Pain in paratibial and posterior tibial perforator veins was present in 95% of patients for whom this information was recorded (519 paratibial and 516 posterior tibial), whereas Doppler ultrasound revealed involvement of the saphenous vein in only 9.1% (46/538) of cases. Other health issues examined included psychological impact related to eating disorders. The SCOFF test (0–5) showed a median score of 2 [IQR: 1–3], while the EAT-40 questionnaire (0–120) yielded a median of 21 [IQR: 14–30].

## 4. Discussion

The results of this study derive from one of the largest published cohorts of patients with lipedema to date (over 1800 women), in which a comprehensive physical examination of signs and symptoms was conducted alongside a detailed anamnesis of comorbidities associated with the disease. Two findings were identified that we consider particularly important.

1.- Since 2020 and throughout the inclusion period (January 2022–November 2024), we have proposed that increased intestinal permeability, accompanied by LPS-induced endotoxemia and potential microbial translocation, acts as an inflammatory trigger for lipedema. Subsequent publications support this hypothesis [42,43]. Given the limited feasibility to perform the physiological gold standard (segmental multi-sugar tests), we operationalized this hypothesis via a clinical questionnaire designed to identify a phenotype compatible with barrier dysfunction. In our cohort, 99% of patients exceeded the prespecified cutoff, suggesting a high burden of compatible symptoms and supporting this idea. This questionnaire does not diagnose increased permeability or endotoxemia, nor does it replace physiological testing; however, it provides evidence-based items to approximate the concept of intestinal permeability, which will require formal validation in future studies. These results are consistent with the high prevalence of increased intestinal permeability and its link to chronic low-grade inflammation, which could be associated with, lipedema. However, in no case is suspected high intestinal permeability mentioned as a cause or etiology of lipedema, but rather high intestinal permeability could be associated with lipedema, although this needs to be demonstrated in studies with longitudinal epidemiological designs with a comparator group to establish its causality.

2.- In this study, nearly all patients were found to have ligamentous hyperlaxity, with a high rate of positivity on the Beighton test (95.8% of 1726 patients).

In addition to the Beighton score, we assessed accessory signs of ligamentous hyperlaxity supported by literature: digital pressure pain over the iliotibial band/TFL (fascial alterations in hEDS), bilateral trochanteric pain (GTPS) with a high prevalence of hypermobility, and lateral ankle instability with recurrent torsion without sprain due to ATFL/CFL laxity. These signs showed high positivity (ITB/TFL 96%, trochanteric pain 97%, recurrent torsion 61%), consistent with the literature on biomechanics and laxity in HSD/hEDS and CAI [44,45,46,47,48,49,50]. This may suggest that lipedema is accompanied by connective tissue impairment, which could be one of its triggering factors. Evidence indicates that the onset of lipedema occurs after the onset of puberty [51], consistent with its recognized hormonally influenced pattern, although childhood connective-tissue features such as generalized hypermobility—and, theoretically, deep-fascia–related perforator dysfunction—may precede the clinical expression of the disease. Connective tissue damage may likewise account for the high percentage of patients in our cohort who presented with pain upon digital pressure of the pre- and paratibial perforators, likely due to impaired drainage with pathological centrifugal flow into the GSV. In our clinical experience, perforators in the femoral canal (Dodd) and adductor canal (Hunter) also elicit pain on digital pressure (unpublished data). Exploration of these tender points is not usually performed in routine clinical practice for diagnosing chronic venous insufficiency (CVI), where ultrasound is the main diagnostic tool. Remarkably, despite the fact that nearly all patients in our study exhibited pain upon examination of the perforators, only 9.1% of the 538 women who underwent Doppler ultrasound of the GSV were diagnosed with this condition (CVI). We hypothesize that the antireflux closure mechanism of the perforator veins that penetrate the muscular fascia obliquely [52,53], including both the Doppler-visible perforators and the many small, non-visible perforators [54], the smallest being valveless [55], is entirely or partially dependent on stretching of the muscular fascia [52,56]. This mechanism may fail as a consequence of connective tissue damage associated with lipedema, leading to a pathological but subclinical outward flow that cannot be detected by Doppler ultrasound [57,58,59].

Another noteworthy finding is that women with more extensive lipedema showed evidence of lymphatic involvement, as indicated by positive ultrasound of a Stone-paved sign in the lower pretibial zone, similar to those observed in lymphedema [60,61,62], though less pronounced on ultrasound. In our experience, they are seen in the distal pretibial region in a supraperiosteal location.

In our study, ultrasound and elastography were used exclusively as non-diagnostic tools. According to the other authors (Faerber G eta al [63] and Hirsch et al. [64]), ultrasonography cannot diagnose lipedema nor distinguish lipedema tissue from obesity. Following the anatomical description by Caggiati [65], we examined the adipose layer within the saphenous compartment (SphSAT). Strain and shear-wave elastography demonstrated markedly increased stiffness of SphSAT, in several cases approaching muscular values. In contrast, the deep subcutaneous adipose tissue (DSAT) was substantially more elastic, consistent with its physiological role as the primary energy-storage depot in women. The superficial pertrochanteric layer (SSAT) also showed reduced elasticity. These biomechanical differences are non-specific and were used only for exploratory characterization. We currently lack a sufficient number of healthy controls to define elastographic reference values. The disproportionate SphSAT thickness described by Caggiati [65] and the elevated stiffness we observed in SphSAT and SSAT—contrasting with the higher elasticity of DSAT—should be interpreted as non-specific mechanical changes. Consistent with S2k recommendations, neither ultrasound echotexture nor elastographic stiffness can differentiate lipedema from obesity, and in this study, they were used strictly for descriptive biomechanical purposes.

We also observed that in nearly 40% of women in our study, lipedema was diagnosed at later ages (perimenopause, menopause, or postmenopause), suggesting a significant diagnostic delay. This finding is consistent with several previous studies, such as the 10-year retrospective cohort by Ghods M. et al. [66], which reported substantial delays in both diagnosis and treatment initiation, in some cases extending up to 18 years from the onset of symptoms, as described by Romeijn JRM [67]. Similarly, in the study by Falck et al. [10], approximately 70% of women experienced symptom onset before the age of 30, yet only three patients (1.6%) received a diagnosis prior to that age. In the Spanish cohort, more than 40% of women were classified as having advanced stages of the disease (Schingale classification types IV and V), indicating that the condition was already highly progressed at the time of diagnosis, with all the associated consequences of poorer physical health and greater limitations in daily life [10]. Altogether, these findings underscore the urgent need for early and timely diagnosis of lipedema, beginning at the primary care level, by family and community physicians, and extending to other specialists consulted for the multiple associated comorbidities.

Regarding comorbidities, inflammatory ovarian dysfunction was diagnosed in 76% of patients, a figure markedly higher than the prevalence of polycystic ovary syndrome reported in the Italian cohort by Patton et al. [68], which was 17.1%. Thyroid disease was identified in more than half of the patients, also well above the prevalence reported by Patton et al. [68], where 22.5% of patients had hypothyroidism. When thyroid involvement was assessed by ultrasound findings, the prevalence was even higher, with thyroid nodules observed in 88.7% of patients. These discrepancies across studies may partly reflect variability in the definitions employed; however, the differences remain striking. It has been suggested that increased intestinal permeability and the resulting state of chronic low-grade inflammation may contribute to the development of autoimmune diseases [69,70,71,72,73].

In future clinical studies on lipedema, both thyroid ultrasound and Doppler imaging should be systematically performed. These assessments should also be considered in the pre-surgical setting.

Another noteworthy finding is the high prevalence of nickel skin allergies among our patients, which should not be confused with systemic allergies. Many patients reported that, both in childhood and currently, wearing costume jewelry led to irritation of the earlobe or other areas in contact with nickel-containing objects. Although this condition could only be assessed in a relatively small subset of our cohort, the prevalence observed was substantially higher than that reported in the general population, where estimates are around 24% [74].

Another comorbidity frequently observed in these patients is related to mental health. Patients demonstrated a high risk of developing eating disorders, findings consistent with previous reports indicating that women with lipedema have higher rates of anxiety, depression, and eating disorders, particularly in the more advanced stages of the disease [15]. These conditions appear to develop as compensatory mechanisms, largely due to the frequent misclassification of lipedema as obesity [75].

In the present study, we identified signs and symptoms of lipedema in a cohort of more than 1800 patients. However, given the lack of a healthy control group, it is not possible to establish definitive diagnostic criteria for the disease. Nevertheless, considering the very high prevalence of these signs and symptoms (ranging from 88% to 98%) in such a large number of diagnosed patients, we propose that the absence of these findings may help to rule out lipedema.

Regarding study limitations, the study design itself does not allow for establishing causal or prognostic relationships in lipedema; however, it does enable the generation of causal hypotheses by describing the distribution of a large number of symptoms, signs, and comorbidities associated with lipedema in a sizable patient cohort. Another limitation of the present study is the lack of validated questionnaires for assessing intestinal permeability and inflammatory ovarian dysfunction. The IODI was developed after many years of clinical observation in women with lipedema, in whom a recurrent pattern of dysmenorrhea, heavy or irregular menses and later ovarian–uterine findings were frequently reported, often leading to the prescription of estrogen–progestin contraceptives for symptom control rather than contraception. This pattern is non-specific and may overlap with PCOS or endometriosis. Experimental and clinical data in endometriosis suggest that chronic inflammation can promote progesterone resistance and a relative estrogen-dominant milieu via altered progesterone receptor signaling and NF-κB–driven pathways. In our study, the IODI should therefore be interpreted only as an exploratory marker of inflammatory ovarian–uterine dysfunction, and any putative link with intestinal hyperpermeability must be considered hypothesis-generating. The IODI is a non-validated, non-specific exploratory instrument. It cannot distinguish between PCOS, endometriosis or other gynecologic conditions and should not be interpreted as a diagnostic marker of intestinal hyperpermeability. The proposed link between chronic low-grade inflammation, progesterone resistance and ovarian dysfunction in lipedema requires prospective validation with standardized endocrine and gynecologic assessment [76,77,78,79,80]. Similarly, the questionnaire to identify patients with suspected high intestinal permeability does not allow for the diagnosis of intestinal hyperpermeability syndrome in any case, as it is still under development and requires criterion and construct validation using a gold standard, such as the lactulose-mannitol test, which is not routinely performed in any of the clinics of the Instituto del Lipedema y la Mujer. Another potential limitation of this study is the lack of validation of the physical signs included in the physical examination. Given that this is an emerging disease with limited clinical knowledge, robust and reliable gold standards are needed to determine criterion validity and other parameters such as sensitivity, specificity, predictive values, and intra- and interprofessional reliability. Despite these limitations, the results are highly promising, as they provide, for the first time, a comprehensive description of findings related to involvement of the saphenous compartment, connective tissue alterations, and suspected high intestinal permeability as possible associated mechanisms of the disease. These findings underscore the need for continued research on this important health issue, using observational and controlled study designs to further understand the disease, its risk factors, prognostic indicators, and the efficacy and effectiveness of treatments to improve disease progression and the quality of life of affected patients.

## Figures and Tables

**Table 1:** Areas and type of evaluation of lipedema on the patient’s body.

Areas Affected by Lipedema	According to Clinic	Lipedematous Involvement. Ultrasound Sign of a “Snowstorm”	Elastographic Involvement of the Saphenous Compartment Greater Decrease in Elasticity	Lymphatic Involvement Ultrasound Sign (Stone-Paved Sign)
Infraumbilical abdomen	Not applicable	Yes/No	Not applicable	Not applicable
Hips	Yes/No	Yes/No	Not applicable	Not applicable
Upper half of thigh	Yes/No	Yes/No	Yes/No	Yes/No
Lower half of thigh	Yes/No	Yes/No	Yes/No	Yes/No
Upper half of the calf	Yes/No	Yes/No	Yes/No	Yes/No
Lower half of the calf	Yes/No	Yes/No	Yes/No	Yes/No
Proximal half of the arm	Yes/No	Not applicable	Not applicable	Not applicable
Distal half of the arm (forearm)	Yes/No	Not applicable	Not applicable	Not applicable
Feet below the ankle	Yes/No	Not applicable	Not applicable	Yes/No

**Table 2:** General Description and Physical Examination of Patients with Lipedema.

Variables	Total (*n* = 1803)
**Sociodemographic**	
Age. mean (SD)	42.9 (11.3)
**Anthropometric**	15.8 [6–11]
Weight (kg), mean (SD)	75 (16.4)
Height (cm), mean (SD)	162 (6.3)
Body Mass Index (kg/m^2^), mean (SD)	28.6 (6.2)
**Obesity**	
Normal weight, *n* (%)	576 (31.9%)
Overweight type 1, *n* (%)	273 (15.1%)
Overweight type 2, *n* (%)	288 (16%)
Obesity type 1, *n* (%)	382 (21.2%)
Obesity type 2, *n* (%)	182 (10.1%)
Obesity type 3 (Morbid), *n* (%)	90 (5%)
Obesity type 4 (Extreme), *n* (%)	8 (0.4%)
Underweight, *n* (%)	4 (0.2%)
**Fat distribution pattern**	
Android, *n* (%)	220 (12.2%)
Gynoid, *n* (%)	1583 (87.8%)
**Bioimpedance analysis**	
Total Body Fat Mass (%), mean (SD)	27.7 (11.8)
Percentage of body fat relative to total body weight (%), mean (SD)	34.4 (9.8)
Percentage of fat in the lower right limb (%), mean (SD)	5.2 (2.1)
Percentage of body weight of the lower right limb (%), mean (SD)	37.5 (9.2)
Percentage of fat in the lower left limb (%), mean (SD)	5.1 (2)
Percentage of body weight of the lower left limb (%), mean (SD)	37.5 (9.3)
**Physical examination**	
Deep pressure pain in the Great Saphenous Compartment (upper half), *n* (%)	1780 (98.7%)
Deep pressure pain in the Great Saphenous Compartment (lower half), *n* (%)	1775 (98.4%)
Deep pressure pain in the Small Saphenous Compartment, *n* (%)	1738 (96.4%)
Superficial pressure pain in the Post. Brachial Compartment, *n* (%)	1443 (80%)
Tender points	
Internal supracondylar, *n* (%)	1781 (98.8%)
Internal infracondylar, *n* (%)	1785 (99%)
Internal supramalleolar, *n* (%)	1750 (97.1%)
Supraolecranon, *n* (%)	1474 (81.8%)
False puncture Internal supracondylar, *n* (%)	1758 (97.5%)
False puncture Internal supramalleolar, *n* (%)	1730 (96%)
False puncture Supraolecranian, *n* (%)	1411 (78.3%)
Loss of fine sensation at the pertrochanteric level, *n* (%) (*n* = 195)	176 (90.3%)
Kaposi-Stemmer sign	
Positive, *n* (%)	138 (7.7%)
Equivocal, *n* (%)	63 (3.5%)
Internal supramalleolar edema, *n* (%) (*n* = 256)	229 (89.5)
Internal supramalleolar pitting edema, *n* (%) (*n* = 1749)	97 (5.5%)
Pretibial edema, *n* (%) (*n* = 293)	259 (88.4%)
Pretibial pitting edema, *n* (%) (*n* = 270)	156 (57.8%)

SD: Standard Deviation.

**Table 3:** Correlations between saphenous compartment signs, obesity, fat distribution pattern, age, and weight.

		Obesity	Fat Distribution Pattern	GSC Pain	ISC Pain	SSC Pain	BC Pain	ISC Point	IIC Point	ISM Point	SO Point	IS False Puncture	IM False Puncture	SO False Puncture	PFS Loss	Weight	Age
Obesity	r	1	−0.315 **	0.061 **	0.062 **	0.087 **	0.251 **	0.044	0.053 *	0.075 **	0.237 **	0.048 *	0.056 *	0.230 **	0.063	0.890 **	0.181 **
	*p*		0.000	0.009	0.008	0.000	0.000	0.062	0.024	0.001	0.000	0.043	0.017	0.000	0.380	0.000	0.000
Fat distribution pattern	r	−0.315 **	1	0.018	−0.033	−0.045	−0.097 **	0.020	−0.003	−0.006	−0.105 **	−0.005	0.018	−0.084 **	0.008	−0.258 **	−0.311 **
	*p*	0.000		0.440	0.161	0.054	0.000	0.385	0.891	0.811	0.000	0.827	0.438	0.000	0.906	0.000	0.000
GSC pain	r	0.061 **	0.018	1	0.545 **	0.346 **	0.141 **	0.708 **	0.635 **	0.444 **	0.125 **	0.362 **	0.303 **	0.096 **	0.219 **	0.054 *	−0.032
	*p*	0.009	0.440		0.000	0.000	0.000	0.000	0.000	0.000	0.000	0.000	0.000	0.000	0.002	0.022	0.172
ISC pain	r	0.062 **	−0.033	0.545 **	1	0.525 **	0.139 **	0.517 **	0.619 **	0.636 **	0.173 **	0.354 **	0.429 **	0.129 **	−0.024	0.033	0.010
	*p*	0.008	0.161	0.000		0.000	0.000	0.000	0.000	0.000	0.000	0.000	0.000	0.000	0.743	0.157	0.667
SSC pain	r	0.087 **	−0.045	0.346 **	0.525 **	1	0.264 **	0.328 **	0.396 **	0.503 **	0.252 **	0.253 **	0.350 **	0.234 **	0.099	0.091 **	0.008
	*p*	0.000	0.054	0.000	0.000		0.000	0.000	0.000	0.000	0.000	0.000	0.000	0.000	0.167	0.000	0.743
BC pain	r	0.251 **	−0.097 **	0.141 **	0.139 **	0.264 **	1	0.083 **	0.131 **	0.156 **	0.885 **	0.116 **	0.151 **	0.857 **	0.053	0.223 **	0.064 **
	*p*	0.000	0.000	0.000	0.000	0.000		0.000	0.000	0.000	0.000	0.000	0.000	0.000	0.461	0.000	0.006
ISC point	r	0.044	0.020	0.708 **	0.517 **	0.328 **	0.083 **	1	0.599 **	0.514 **	0.117 **	0.371 **	0.259 **	0.101 **	−0.024	0.048 *	−0.001
	*p*	0.062	0.385	0.000	0.000	0.000	0.000		0.000	0.000	0.000	0.000	0.000	0.000	0.743	0.041	0.965
IIC point	r	0.053 *	−0.003	0.635 **	0.619 **	0.396 **	0.131 **	0.599 **	1	0.506 **	0.140 **	0.485 **	0.404 **	0.109 **		0.052 *	0.000
	*p*	0.024	0.891	0.000	0.000	0.000	0.000	0.000		0.000	0.000	0.000	0.000	0.000	0.000	0.027	0.997
ISM point	r	0.075 **	−0.006	0.444 **	0.636 **	0.503 **	0.156 **	0.514 **	0.506 **	1	0.195 **	0.306 **	0.509 **	0.152 **	0.196 **	0.063 **	0.039
	*p*	0.001	0.811	0.000	0.000	0.000	0.000	0.000	0.000		0.000	0.000	0.000	0.000	0.006	0.007	0.098
SO point	r	0.237 **	−0.105 **	0.125 **	0.173 **	0.252 **	0.885 **	0.117 **	0.140 **	0.195 **	1	0.099 **	0.143 **	0.827 **	0.029	0.210 **	0.074 **
	*p*	0.000	0.000	0.000	0.000	0.000	0.000	0.000	0.000	0.000		0.000	0.000	0.000	0.682	0.000	0.002
IS False puncture	r	0.048 *	−0.005	0.362 **	0.354 **	0.253 **	0.116 **	0.371 **	0.485 **	0.306 **	0.099 **	1	0.544 **	0.122 **	−0.041	0.030	0.024
	*p*	0.043	0.827	0.000	0.000	0.000	0.000	0.000	0.000	0.000	0.000		0.000	0.000	0.569	0.204	0.311
IM False puncture	r	0.056 *	0.018	0.303 **	0.429 **	0.350 **	0.151 **	0.259 **	0.404 **	0.509 **	0.143 **	0.544 **	1	0.199 **	0.093	0.042	0.009
	*p*	0.017	0.438	0.000	0.000	0.000	0.000	0.000	0.000	0.000	0.000	0.000		0.000	0.198	0.075	0.708
SO False puncture	r	0.230 **	−0.084 **	0.096 **	0.129 **	0.234 **	0.857 **	0.101 **	0.109 **	0.152 **	0.827 **	0.122 **	0.199 **	1	0.049	0.202 **	0.071 **
	*p*	0.000	0.000	0.000	0.000	0.000	0.000	0.000	0.000	0.000	0.000	0.000	0.000		0.498	0.000	0.003
PFS Loss	r	0.063	0.008	0.219 **	−0.024	0.099	0.053	−0.024	---	0.196 **	0.029	−0.041	0.093	0.049	1	0.120	0.006
	*p*	0.380	0.906	0.002	0.743	0.167	0.461	0.743	0.000	0.006	0.682	0.569	0.198	0.498		0.096	0.937
Weight	r	0.890 **	−0.258 **	0.054 *	0.033	0.091 **	0.223 **	0.048 *	0.052 *	0.063 **	0.210 **	0.030	0.042	0.202 **	0.120	1	0.101 **
	*p*	0.000	0.000	0.022	0.157	0.000	0.000	0.041	0.027	0.007	0.000	0.204	0.075	0.000	0.096		0.000
Age	r	0.181 **	−0.311 **	−0.032	0.010	0.008	0.064 **	−0.001	0.000	0.039	0.074 **	0.024	0.009	0.071 **	0.006	0.101 **	1
	*p*	0.000	0.000	0.172	0.667	0.743	0.006	0.965	0.997	0.098	0.002	0.311	0.708	0.003	0.937	0.000	

GSC: Great saphenous compartment; ISC pain: Inferior saphenous compartment; SSC: Small Saphenous Compartment; BC: Brachial Compartment; ISC point: Internal supracondylar; IIC: Internal infracondylar; ISM: Internal supramalleolus; SO: Supraolecranon; IS: Internal supracondyle; IM: Internal malleolar; PFS: Pertrochanteric fine sensitivity; r: Pearson’s correlation coefficient; *p*: Level of statistical significance. **. The correlation is significant at *p* < 0.01 (2-tailed). *. The correlation is significant at the 0.05 level (2-tailed). ---. Absence of data due to lack of entries for one of the variable’s categories.

**Table 4:** Classification and diagnosis of lipedema.

Variables	Total
**Classification of Schingale, *n* (%) (*n* = 1796)**	
Type I	290 (1.6%)
Type II	392 (21.8%)
Type III	539 30%)
Type IV	768 (42.8%)
Type V	68 (3.8%)
**Classification of Schmeller, *n* (%) (*n* = 1792)**	
Stage I	799 (44.6%)
Stage II	674 (37.6%)
Stage III	233 (13%)
**Clinical diagnosis, *n* (%) (*n* = 1802)**	
Hips	1787 (99.2%)
Upper thigh	1787 (99.2%)
Lower thigh	1780 (98.8%)
Upper calf	1640 (91%)
Lower calf	1557 (86.4%)
Proximal arm	899 (49.9%)
Distal arm	90 (5%)
Feet below the ankle, (*n* = 1693)	21 (1.2%)
**Diagnosis based on the “Snowstorm” ultrasound sign, *n* (%) (*n* = 1801)**	
Abdomen	2 (0.1%)
Hips	11 (0.6%)
Upper thigh	1765 (97.9%)
Lower thigh	1761 (97.7%)
Upper calf	1728 (95.9%)
Lower calf	1700 (94.3%)
**Diagnosis based on elastographic involvement of the great saphenous vein compartment, *n* (%) (*n* = 1802)**	
Upper thigh	1789 (99.3%)
Lower thigh	1761 (97.7%)
Upper calf	1356 (75.2%)
Lower calf	1119 (62.1%)
**Diagnosis based on the “Stone-paved sign” ultrasound sign, *n* (%) (*n* = 1802)**	
Upper thigh	2 (0.1%)
Lower thigh	1 (0.1%)
Upper calf	164 (9.1%)
Lower calf	851 (47.2%)
Feet below the ankle	125 (6.9%)

**Table 5:** Correlations between the Stone-paved sign, lipedema classifications, age and weight.

		Stone-Paved Sign UT	Stone-Paved Sign LT	Stone-Paved Sign UC	Stone-Paved Sign LC	Stone-Paved Sign Below the Ankle	Classification of Schingale	Classification of Schmeller	Age	Weight
Stone-paved sign UT	r	1	−0.001	−0.011	0.002	−0.009	0.028	−0.011	0.020	0.003
	*p*		0.973	0.655	0.937	0.699	0.237	0.640	0.408	0.893
Stone-paved sign LT	r	−0.001	1	−0.007	0.025	−0.006	0.020	0.032	−0.023	0.055 *
	*p*	0.973		0.752	0.291	0.785	0.403	0.183	0.334	0.020
Stone-paved sign UC	r	−0.011	−0.007	1	0.319 **	0.410 **	0.221 **	0.224 **	0.107 **	0.284 **
	*p*	0.655	0.752		0.000	0.000	0.000	0.000	0.000	0.000
Stone-paved sign LC	r	0.002	0.025	0.319 **	1	0.272 **	0.376 **	0.287 **	0.147 **	0.353 **
	*p*	0.937	0.291	0.000		0.000	0.000	0.000	0.000	0.000
Stone-paved sign below the ankle	r	−0.009	−0.006	0.410 **	0.272 **	1	0.123 **	0.173 **	0.027	0.176 **
	*p*	0.699	0.785	0.000	0.000		0.000	0.000	0.257	0.000
Classification of Schingale	r	0.028	0.020	0.221 **	0.376 **	0.123 **	1	0.388 **	0.167 **	0.487 **
	*p*	0.237	0.403	0.000	0.000	0.000		0.000	0.000	0.000
Classification of Schmeller	r	−0.011	0.032	0.224 **	0.287 **	0.173 **	0.388 **	1	0.198 **	0.541 **
	*p*	0.640	0.183	0.000	0.000	0.000	0.000		0.000	0.000
Age	r	0.020	−0.023	0.107 **	0.147 **	00.027	0.167 **	0.198 **	1	0.094 **
	*p*	0.408	0.334	0.000	0.000	0.257	0.000	0.000		0.000
Weight	r	0.003	0.055 *	0.284 **	0.353 **	0.176 **	0.487 **	0.541 **	0.094 **	1
	*p*	0.893	0.020	0.000	0.000	0.000	0.000	0.000	0.000	

UT: upper thigh; LT: lower thigh; UC: upper calf; LC: lower calf; r: Pearson correlation; *p*: statistical significance level. *. The correlation is significant at the 0.05 level (two-tailed). **. The correlation is significant at the 0.01 level (two-tailed).

**Table 6:** Comorbidities in lipedema.

Variables	Total
Suspected high intestinal permeability, *n* (%)	1785 (99%)
Ligamentous hyperlaxity syndrome, *n* (%) (*n* = 1801)	1726 (95.8%)
Beighton test (0–9), media (DE)	7.5 (1.6)
Bilateral trochanteric pain region, *n* (%) (*n* = 1799)	1753 (97.4%)
Iliotibial band involvement (Tensor fascia lata), *n* (%) (*n* = 1799)	1737 (96.6%)
Non-dislocated, recurrent ankle sprains due to mechanical instability, *n* (%) (*n* = 1798)	1106 (61.5%)
Nickel allergy, *n* (%) (*n* = 130)	88 (67.7%)
Thyroid pathology, *n* (%) (*n* = 1801)	1073 (59.5%)
Type of thyroid pathology, *n* (%) (*n* = 533)	
Thyroid nodules	473 (88.7%)
Multinodular goitre	46 (8.6%)
Hypothyroidism	13(2.4%)
Hashimoto’s syndrome	1 (0.2%)
Inflammatory ovarian dysfunction, *n* (%) (*n* = 1801)	1368 (76%)
Great saphenous vein, on Doppler ultrasound observation, *n* (%) (*n* = 538)	46 (9.1%)
Pain in paratibial perforators, *n* (%) (*n* = 543)	519 (95.6%)
Pain in post-tibial perforators, *n* (%) (*n* = 538)	516 (95.9%)
Treatment with psychologist, *n* (%) (*n* = 531)	329 (62%)
Scoff test (0–5), median [p25–p75]	2 [1–3]
Test EAT-40 (0–120), median [p25–p75]	21 [14–30]

EAT: Eating Attitudes Test.

## Data Availability

Data presented in this study is contained within the article and Supplementary Materials. Further inquiries can be directed to the corresponding author.

## Supplementary Materials

The following supporting information can be downloaded at: https://www.mdpi.com/article/10.3390/biomedicines13123049/s1, Table S1: ILM Intestinal Hyperpermeability Questionnaire; Table S2: Beighton Test; Table S3: Exploratory Questionnaire: Inflammatory Ovarian Dysfunction Index (IDOI); Table S4: Eating Attitudes Test-40; Table S5: Scoff Questionnaire; Table S6: Schingale Classification; Table S7: Classification Schmeller.
