# Supplementary material for: Clinical Signs at Diagnosis and Comorbidities in a Large Cohort of Patients with Lipedema in Spain
Source: Biomedicines. 2025 Dec 11;13(12):3049. doi: 10.3390/biomedicines13123049 (PMC12731049; doi:10.3390/biomedicines13123049)
Supplement: Supplementary file 1 [file biomedicines-13-03049-s001.zip › biomedicines-3944375-supplementary.pdf]

Supplementary material

Table S1. ILM Intestinal Hyperpermeability Questionnaire

1. Do you notice a feeling of exhaustion (not tiredness) at night or throughout the day?	14. Do you have an allergy to nickel (earrings/rings) or others (dust mites, grasses, dander, or pollen), asthma, rhinitis, sinusitis, or atopy?
2. Do you almost always fall asleep while watching TV in the afternoon/evening? (even very short episodes).	15. Do you sometimes get temporary redness on your chest, neck, or face?
3. Do you have trouble falling asleep and wake up often?	16. Do you frequently have headaches, and if you are a woman, especially with menstruation?
4. Do you often feel sensations of heat and/or cold without having a fever? (Chills).	17. Do you have many spider veins, or medium to large varicose veins?
5. Do you frequently (at least twice a month) feel like you are “coming down” with an illness?	18. Do you frequently have episodes of anemia, vitamin D deficiency, or folic acid deficiency?
6. Do you wake up feeling tired?	19. Do you usually feel hungry shortly after eating (especially for flour-based foods and/or sugar)?
7. When you eat lettuce, do you “bloat”? Especially at night (does not happen with small-leaf varieties).	20. Have you ever had an episode of depression, or do you usually experience intense anxiety regularly?
8. Do you often have intestinal movement (noises) in the lower abdomen (Borborygmi)?	21. Do you have erratic and frequent joint and/or muscle pains? (Spine, knees, wrists, ankles, etc.)
9. Do you usually suffer from habitual constipation, or on the contrary, very loose stools?	22. If you are a woman, how were your periods between the ages of 12–20? Painful, irregular, and/or heavy?
10. Do meals generally “repeat” on you when you eat the same as others around you?	23. Do you often have sudden changes (from one day to the next) in your weight or body volume, not related to what you ate the previous day?
11. Do you usually have significant fluid retention in your legs?	24. Have you suffered from plantar fasciitis or carpal tunnel syndrome?
12. Do you notice bloating (swelling) in the lower abdomen that varies greatly from day to day?	25. If at any time in the last five years you followed a non-ketogenic diet, did it happen between the 2nd and 4th week that you stopped losing weight even though you were following the diet correctly?
13. Does cow’s milk upset you?	26. Do you feel that you eat less than others and still gain weight?

Score: One point for each positive answer.
How is the degree of hyperpermeability measured?
The greater the number of positive answers, the greater the hyperpermeability.

- Between 21–26 positive answers – 4 crosses.
- Between 19–20 positive answers – 3 crosses.
- Between 17–18 positive answers – 2 crosses.
- Below 17 positive answers – 1 crosses ▯ Doubtful.

- Losurdo G.; Principi M.; Iannone A.; Amoruso A.; Ierardi E.; Di Leo A.; Barone M. Extra-intestinal manifestations of non-celiac gluten sensitivity: An expanding paradigm. *World J Gastroenterol*. **2018**, 24(14),1521-1530.
- Neroni B.; Evangelisti M.; Radocchia G.; Di Nardo G.; Pantanella F.; Villa M.P.; Schippa S. Relationship between sleep disorders and gut dysbiosis: what affects what? *Sleep Med*. **2021**, 87, 1-7.
- Cardoso-Silva D.; Delbue D.; Itzlinger A.; Moerkens R.; Withoff S.; Branchi F.; Schumann M. Intestinal Barrier Function in Gluten-Related Disorders. *Nutrients*. **2019**, 11(10), 2325.
- Dantzer R.; O'Connor J.C.; Freund G.G.; Johnson R.W.; Kelley K.W. From inflammation to sickness and depression: when the immune system subjugates the brain. *Nat Rev Neurosci*. **2008**, 9(1), 46-56.
- Camilleri M. Leaky gut: mechanisms, measurement and clinical implications in humans. *Gut*. **2019**, 68(8),1516-1526.
- Halmos E.P.; Power V.A.; Shepherd S.J.; Gibson P.R.; Muir J.G.; A diet low in FODMAPs reduces symptoms of irritable bowel syndrome. *Gastroenterology*. **2014**, 146(1):67-75.e5.
- Miglietta S.; Borghini R.; Relucenti M.; Sorrentino V.; Chen R.; Li X.; Fazi F.; et al. New Insights into Intestinal Permeability in Irritable Bowel Syndrome-Like Disorders: Histological and Ultrastructural Findings of Duodenal Biopsies. *Cells*. **2021**, 10(10), 2593.

- Staudacher H.M.; Whelan K. The low FODMAP diet: recent advances in understanding its mechanisms and efficacy in IBS. *Gut*. **2017**, 66(8),1517-1527.
- Lombardi F.; Fiasca F.; Minelli M.; Maio D.; Mattei A.; Vergallo I.; et al. The Effects of Low-Nickel Diet Combined with Oral Administration of Selected Probiotics on Patients with Systemic Nickel Allergy Syndrome (SNAS) and Gut Dysbiosis. *Nutrients*. **2020,** 12(4), 1040.
- Portincasa P.; Bonfrate L.; Khalil M.; Angelis M.; Calabrese F.M.; D'Amato M.; et al. Intestinal Barrier and Permeability in Health, Obesity and NAFLD. *Biomedicines*. **2021**, 10(1), 83.

The Intestinal Permeability Test (IPT©) described in this article is the original creation of Jose Luis Simarro, 2025, and is currently undergoing registration at the Spanish Intellectual Property Office. Any reproduction, modification, or adaptation of the test requires proper citation and prior authorization from the author.

Table S2. Beighton Test

Limb	Beighton points	
	Right	Left
Elbow hyperextension	*	*
Touching the forearm with the thumb	*	*
Passive extension of the index finger	*	*
Hyperextension of the knee	*	*
Anterior flexion of the trunk	*	
Score: Presenting a "positive Beighton score," which requires a score of 4 or more out of a total of 9. Subjects are evaluated on a 9-point scale, with 1 point awarded for each hypermobile joint site, measured bilaterally		

- Beighton P.; Solomon L.; Soskolne CL. Articular mobility in an African population. *Ann Rheum Dis*. **1973**, 32(5),413–418.

Table S3. Exploratory Questionnaire: Inflammatory Ovarian Dysfunction Index (IDOI)

Item	Question	Response Options	Scoring
1	Between the ages of 12 and 20, were your periods so painful that you had to interrupt your activities on the first day?	Yes / No	Yes = 1, No = 0
2	Between the ages of 12 and 20, were your periods very painful and did you need to take analgesics?	Yes / No	Yes = 1, No = 0
3	Between the ages of 12 and 20, were your periods very heavy, especially on the first day?	Yes / No	Yes = 1, No = 0
4	Between the ages of 12 and 20, were your menstrual cycles not 28 days in length?	Yes / No	Yes = 1, No = 0

- Zhai J.; Vannuccini S.; Petraglia.; F. Giudice L.C. Adenomyosis: Mechanisms and Pathogenesis. *Semin Reprod Med*. **2020**, 38(2-03), 129-143.
- Jain V.; Chodankar R.R.; Maybin J.A.; Critchley HOD. Uterine bleeding: how understanding endometrial physiology underpins menstrual health. Nat Rev Endocrinol. 2022, 18(5), 290-308.
- Velez L.M.; Seldin M.; Motta A.B. Inflammation and reproductive function in women with polycystic ovary syndrome†. *Biol Reprod*. **2021**, 104(6), 1205-1217.

Table S4. Eating Attitudes Test-40

1. I like eating with other people	21. I have difficulty swallowing.
2. I prepare meals for others, but I don't eat them myself.	22. I cut my food into very small pieces.
3. I get nervous when mealtimes approach	23. I feel very bad after eating sweets.
4. I am very afraid of weighing too much.	24. I lie to others about what I eat.
5. I try not to eat even when I am hungry.	25. I like to try new recipes.
6. I am preoccupied with wanting to be thinner.	26. It bothers me when others encourage me to eat.
7. I have specific rules about food.	27. I am afraid of being unable to stop eating once I start.
8. I believe that food dominates my life.	28. I vomit if I feel heavy.
9. I like the feeling of having an empty stomach.	29. I feel that others pressure me to eat.
10. I dislike the feeling of being full.	30. I eat slowly.
11. I am terrified of gaining weight.	31. I feel like my stomach is always empty.
12. I spend too much time thinking about food.	32. I have obsessive thoughts about food.
13. I feel disgusted after eating.	33. I eat alone.
14. I feel very guilty after eating.	34. I eat out of anxiety.
15. I am able to control what I eat.	35. I eat in secret.
16. I weigh myself several times a day.	36. I feel out of control when I eat.
17. I am interested in knowing how much other people weigh.	37. I am afraid to eat in public places.
18. I avoid eating when I am alone.	38. I think other people are watching me when I eat.
19. I vomit after eating.	39. I feel uncomfortable if other people see me eating.
20. I dislike eating sweets.	40. I feel like vomiting after meals.
**Answer**	**Score**
Always	3
Almost always	2
Quite often	1
Sometimes	0
Almost never	0
Never	0

- Castro J.; Toro J.; Salamero M.; Guimerá E. The Eating Attitudes Test: validation of the Spanish version. *Evaluación Psicológica/Psychol. Assess.* **1991**, *7*, 175–190.

Table S5. Scoff questionnaire

Questions	Yes	No
Do you feel ill because your stomach feels so full that it is uncomfortable?		
Are you worried because you feel you have to control how much you eat?		
Have you recently lost more than 6 kg in a three-month period?		
Do you think you are fat even though others say you are too thin?		
Would you say that food dominates your life?		
TOTAL SCORE (1 point for each YES)		

- Garcia-Campayo J.; Sanz-Carrillo C.; Ibañez J.A.; Lou S.; Solano V, Alda M. Validation of the Spanish version of the SCOFF questionnaire for the screening of eating disorders in primary care. *J Psychosom Res*. **2005**, 59(2), 51-5.

Table S6. Schingale Classification

The anatomical classification most widely used in clinical practice was originally proposed by Schingale in 2003, who described five types of lipedema according to the distribution of abnormal adipose tissue (I: buttocks and hips; II: buttocks to knees; III: buttocks to ankles; IV: legs and arms; V: predominantly lower legs). This system has since been adopted and refined in subsequent German and European guidelines as the standard morphological classification of lipedema.

Classification	Description
Type I	The adipose tissue is increased on buttocks and thighs.
Type II	Lipoedema extends to the knees, especially on the inner side.
Type III	Lipoedema extends from the hips to the ankles.
Type IV	Lipoedema affects the arms and legs.
Type V	Lipedema mainly affects the area from the knee to the ankle, although the upper half of the leg may be slightly affected.

- Schingale F. Lymphödeme – Lipödeme: Diagnose und Therapie. Ein Ratgeber für Betroffene. Hannover: Schlütersche Verlagsgesellschaft. **2003**, 176 S.

Table S7. Classification Schmeller

Classification	Classification
Grade I	The skin is smooth, but the subcutaneous tissue is thickened and, when palpated, small, soft, non-encapsulated nodules are noticeable.
Grade II	Irregularly sized nodules (ranging from the size of a walnut to an apple) appear unencapsulated in the subcutaneous tissue. They are hard in consistency and protrude from the skin.
Grade III	The fat deposits are even larger and clearly deform the limb. This is often associated with lipolymphedema.

- Reich-Schupke S.; Schmeller, W.; Brauer, W.J.; Cornely, M.E.; Faerber, G.; Ludwig, M.; Lulay, G.; Miller, A.; Rapprich, S.; Richter, D.F.; et al. S1 guidelines: Lipedema. J. Dtsch. Dermatol. Ges. 2017, 15, 758–767.

Deep Pinch / Pseudo pinch / Pseudo pinch sign (Manual Technique)

Deep pinch: A manual technique in which the examiner grasps the tissue between the thumb and index finger, with the fingers forming a curved arc to engage the deep subcutaneous layer. This allows assessment of tenderness, resistance, and nodularity. It is not performed by pressing the skin with flat fingertips, as this only captures superficial tissue.

False Prick / False Puncture (Needle Sign)

False prick (false puncture): A needle-related sign produced by gently but firmly pressing a sterile needle at approximately 15° on the skin, generating a pricking sensation without true penetration. This technique does not involve sliding the needle over the surface and is entirely distinct from the manual deep pinch technique. It is also advisable to perform the false prick sign on an area of the body not affected by lipedema so that the patient can perceive that, under the same stimulus, no pricking sensation is elicited.
